# Detection and Monitoring of Toxic Chemical at Ultra Trace Level by Utilizing Doped Nanomaterial

**DOI:** 10.1371/journal.pone.0109423

**Published:** 2014-10-16

**Authors:** Sher Bahadar Khan, Mohammed M. Rahman, Kalsoom Akhtar, Abdullah M. Asiri

**Affiliations:** 1 Chemistry Department, Faculty of Science, King Abdulaziz University, Jeddah, Saudi Arabia; 2 Center of Excellence for Advanced Materials Research (CEAMR), King Abdulaziz University, Jeddah, Saudi Arabia; 3 Division of Nano Sciences and Department of Chemistry, Ewha Womans University, Seoul, Korea; Peking University, China

## Abstract

Composite nanoparticles were synthesized by eco-friendly hydrothermal process and characterized by different spectroscopic techniques. All the spectroscopic techniques suggested the synthesis of well crystalline optically active composite nanoparticles with average diameter of ∼30 nm. The synthesized nanoparticles were applied for the development of chemical sensor which was fabricated by coating the nanoparticles on silver electrode for the recognition of phthalimide using simple I–V technique. The developed sensor exhibited high sensitivity (1.7361 µA.mM^−1^.cm^−2^), lower detection limit (8.0 µM) and long range of detection (77.0 µM to 0.38 M). Further the resistances of composite nanoparticles based sensor was found to be 2.7 MΩ which change from 2.7 to 1.7 with change in phthalimide concentration. The major advantages of the designed sensor over existing sensors are its simple technique, low cost, lower detection limit, high sensitivity and long range of detection. It can detect phthalimide even at trace level and sense over wide range of concentrations. Therefore the composite nanoparticals would be a better choice for the fabrication of phthalimide chemical sensor and would be time and cost substituted implement for environmental safety.

## Introduction

Environmental pollution has acknowledged substantial consideration in recent times due to detrimental upshot on human health and living beings [1]–[3]. The industrial development acts as a source towards several stern environmental dilemmas by liberating plentiful toxic compound into the globe. Accretion of xenobiotics and different hazardous chemicals in soil and water resulted in production of thousands of hazardous waste locations over the years [4]–[6]. Accidental seepage of unsafe chemicals is amongst the main cause regarding environmental pollution. Thus revealing and scrutinizing of perilous chemicals and dilapidation of organic pollutants are vital for environmental pollution control and industrial purposes. Phthalimide is an organic pollutant having toxic nature. Phthalimide is used in plastics, different chemical synthesis, dyes and fungicide. It is toxic material which acts as strong skin, eye and upper respiratory tract irritant. Phthalimide fungicides are widely used in agricultural products. Phthalimide fungicides include captan and captafol which are skin sensitizers and can cause cancer and liver, reproductive, and developmental toxicity to human’s health [7].

Many attempts have been made for the recognition and determination of hazardous chemicals using chromatographic and spectroscopic techniques, but are not significant due to impediment and listlessness [3], [5], [8]. On the other hand, electrochemical sensors are favorable for finding and verification of dicey compounds due to straightforward and fast operation, response and detection [9], [10]. However sensitivity, selectivity, and high cost difficulties related to sensors need improvement in order to make possible their more extensive use [1], [10]. The size, structure and properties of electrode materials determine sensitivity and selectivity of electrochemical sensor [1], [10]. Therefore semiconductor nanostructured materials have obtained much significance and have widely been utilized as a redox mediator in chemical sensors and many other applications [11]–[15].

Manganese and iron oxides with various crystalline structures have been investigated for many purposes especially catalytic and sensing properties [16], [17]. Nonetheless, to develop various properties of manganese and iron oxide in order to meet the growing needs for different functions, it is needed to amend the features of these oxides. Introduction of one nanomaterial into the defects of other material system is one of the major noteworthy means to adjust the texture of the nanostructures because of late doped metal oxide has given away excellent properties in different areas [18], [19].

Therefore, the present study is the continuation of our effort for the development of chemical sensor for environmental and medicinal applications [1], [10]. In this contribution, growth of composite nanoparticles was carried out by simple low cost method and analyzed by XRD, FESEM, EDS, FTIR, and UV-vis. spectroscopy. The synthesized doped nanoparticles were utilized to develop sensor and applied for the recognition of phthalimide at micro level. Further optimization of sensitivity and selectivity of the developed sensor was carried out by studying various parameters. The fabricated sensor may be utilized as chemical sensing element in the devices, which are used for environmental monitoring and industrial applications.

## Experimental Section

### 1. Synthesis and characterization of composite nanoparticles

FeCl_3_ and MnCl_2_ were dissolved in double distilled water in equal mole ratio and then the mixture solution wasprepared high basic (pH 10) by adding NH_4_OH. The basic solution was then heated up to 150.0°C for 15 hours. After 15 hrs, the product was collected by decanting the solvent and washed several times with double distilled water and acetone. The product was dried and then calcined at 500.0°C for 5 hours. The synthesized nanomaterial was characterized by utilizing various spectroscopic methods. Morphology was studied characterize by XRD (X’Pert Explorer, PANalytical diffractometer) and JEOL Scanning Electron Microscope (JSM-7600F, Japan). FT-IR spectrum is taken for the structure confirmation of doped nanomaterial using PerkinElmer (spectrum 100) FT-IR spectrometer. UV spectrum is taken for the optical performance of nanomaterial using PerkinElmer (Lambda 950) UV-visible spectrometer. Keithley Electrometer was utilized for the current measurement.

### 2. Possible growth mechanism of composite nanoparticles

**Figure 1:**
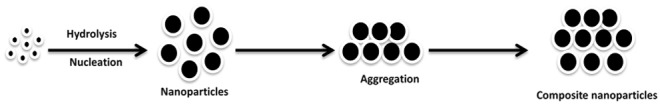
Schematic view for growth mechanism of composite nanoparticles.

The synthesis of composite nanoparticles is carried out by nucleation followed by growth. Initially, MnCl_2_ and FeCl_3_ go through hydrolysis using NH_4_OH and yield Mn^2+^, Fe^3+^ and OH^−^. Mn^2+^ react with OH^−^ and produces Mn(OH)_2_. The heating promotes the dehydration of Mn(OH)_2_ and generate small MnO_3_ nuclei. During growth process (Fig. 1), MnO_3_ react with Fe^3+^, nucleus development occur which then assemble and produce FeMnO_3_ nanoparticles by Ostwald ripening. The nanoparticles form crystals and combine together through Van der Waals forces and hydrogen bonding which provide FeMnO_3_ composite nanoparticles.

### 3. Fabrication and detection technique

**Figure 2:**
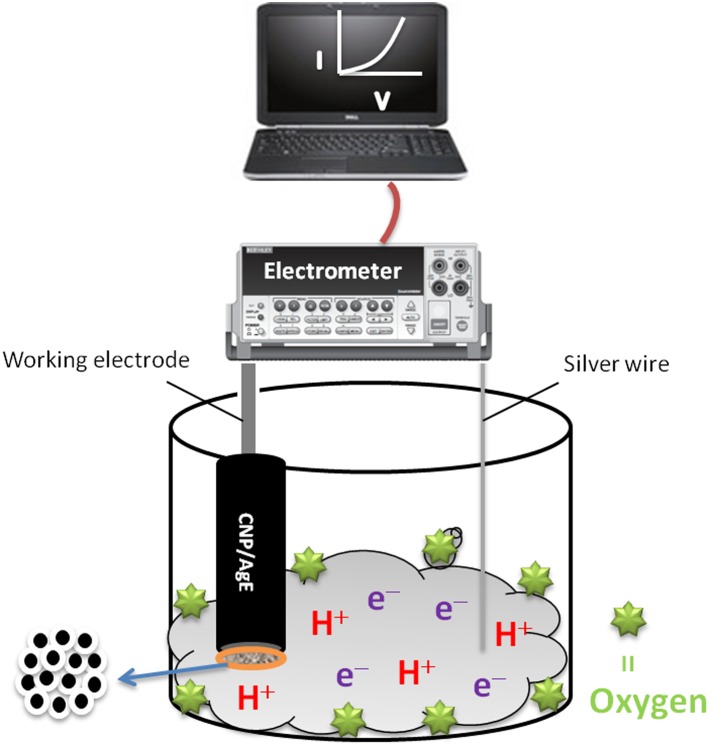
Construction of cell for the detection of phthalimide.

Silver electrode (AgE) was coated with doped nanomaterial along with carbitol and was dried at 60.0°C for 12 hours. The coated AgE was used as a working electrode along with counter electrode (pd wire). Current variation was measured for phthalimide at different micro-level concentration in specific voltage range of 0 to 1.0 V. The delay and response time for the measurement was fixed somehow like 1.0 and 10.0 sec, respectively. All the measurement was carried out in 10.0 ml of 0.1 M buffer having pH 7.0. The constructed cell consists of two electrode, working electrode (Fig. 2) and counter electrode (Pd wire). Detecting material concentration in micro level was prepared in buffer solution as targeting chemical. The modified silver electrode was dipped in different concentration of phthalimide and recorded the sensing properties of doped material by I–V technique. The sensitivity and detection limit was obtained from I–V data. Fig. 2 presents the diagram of the constructed cell for phthalimide sensor.

## Results and Discussion

### 1. Structural and morphological characterization

**Figure 3:**
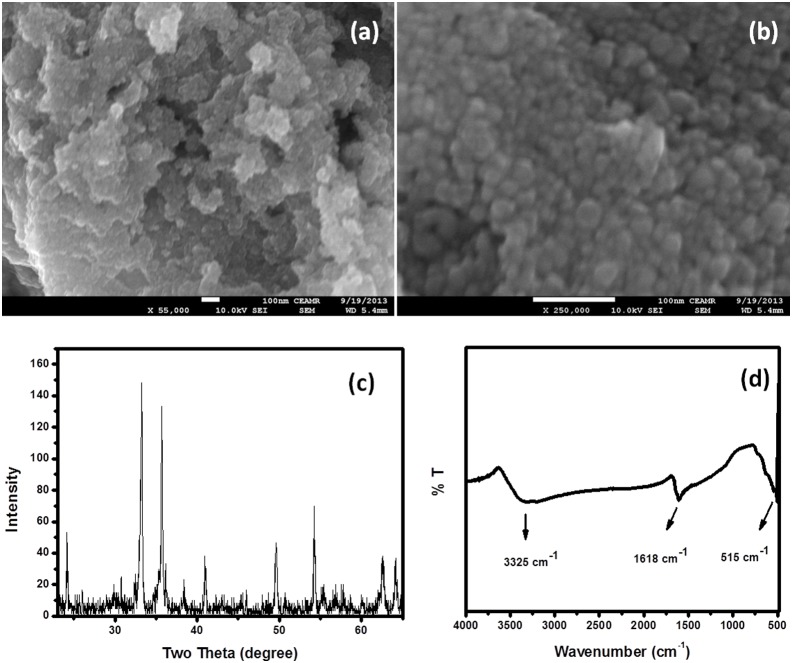
Typical (a) low-magnification and (b) high-resolution FESEM images, (c) typical XRD pattern and (d) FTIR spectrum of composite nanoparticles.

Morphological analysis of the composite material was carried out by FESEM which are shown in Fig. 3 (a, b). FESEM images with different magnification demonstrate that the composite material is grown in particle shape with an average diameter of almost ∼30 nm. The nanoparticles are spherical with uniform distribution in high concentration.

The crystal structure of composite nanoparticles was examined by X-ray powder diffraction and the outcomes are illustrated in Figure 3(c). XRD spectrum of composite nanoparticles exhibited crystalline peaks associated to cubic FeMnO_3_ (JCPDS #76-0076) with a space group of Ia3, lattice constants of *a* = *b* = *c* = 9.3650Å and *α* = *β* = *γ* = 90°. All the attributed peaks are suited with FeMnO_3_. There is no extra impurity peak in XRD spectrum which specifies that the nanoparticles are high crystalline composite of iron and manganese oxide (FeMnO_3_).

The structure and functional group of composite nanoparticles was assessed by FT-IR spectroscopy which is presented in Figure 3(d). FT-IR spectrum is conducted in the wave number of 400∼4000 cm^−1^ and composite nanoparticles showed absorption at 515, 1618 and 3325 cm^−1^. The absorption band appeared at 3325 cm^−1^ and 1618 cm^−1^ are due to O-H stretching and bending respectively which predict the absorption of moisture from environment due to porous nature of composite nanoparticle [1]. FT-IR spectrum of composite nanoparticles also exhibited strong absorption band at 508 cm^−1^ for metal oxygen bond stretching (M–O, M = Mn and Fe) [10].

**Figure 4:**
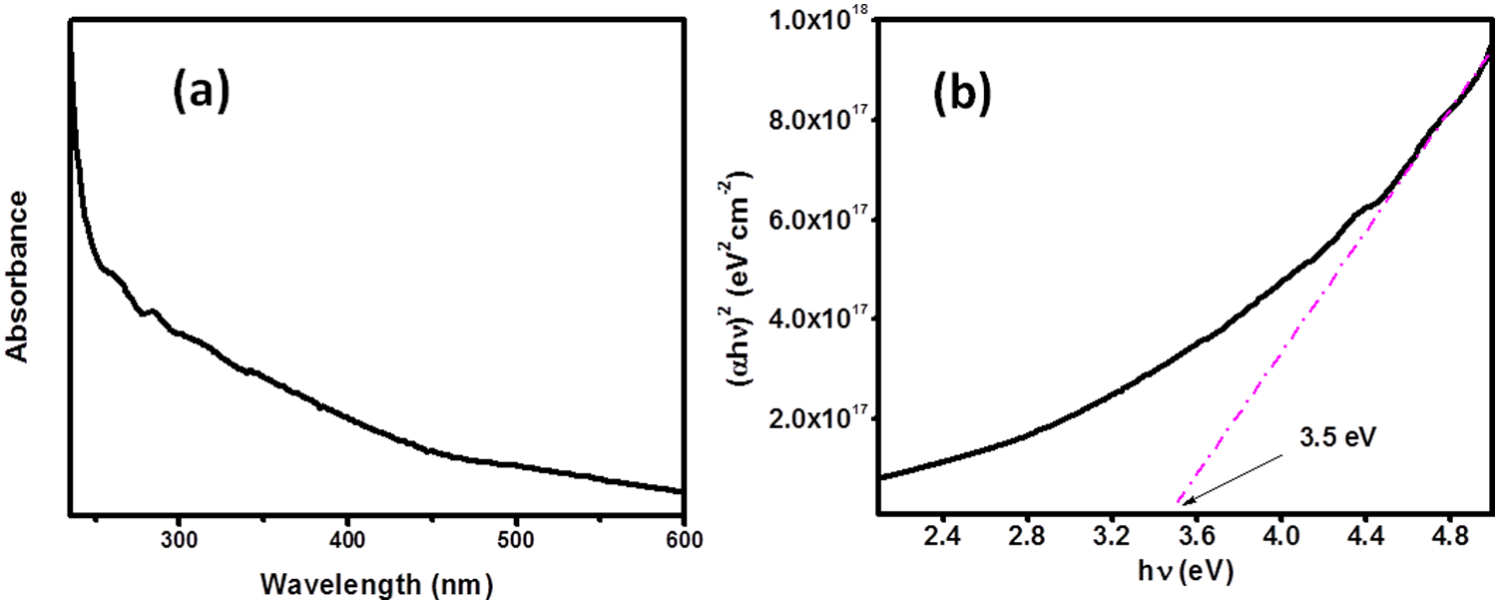
UV spectrum of composite nanoparticles.

The optical asset of the composite nanoparticle was monitored by UV-Visible spectrophotometer and is illustrated in Fig. 4(a). UV–vis absorption spectrum exhibited absorption peak at 354 nm. The band gap energy *E_g_* of composite nanoparticles was originated to be about 3.5 eV from the line drawn at rectilinear plateau of curve *(αhν)^2^* vs. *hν* (Fig. 4(b)).

### 2.2 Chemical sensing properties

#### 2.1. Performance of the phathalimide sensor

From application point of view, fabrication and progress of phthalimide chemical sensor has gained much importance in various fields like medicine, home safety, environmental pollution etc. Therefore, composite nanoparticles were employed to develop a sensor for detecting the presence of phthalimide and its concentrations using I–V technique [18], [19]. This developed chemical sensor contains two basic components i.e. receptor and physicochemical transducer. In the fabricated sensor, composite nanoparticles work as a chemical recognition system (receptor) which intermingles with phthalimide and consequently alter its physical properties in such a manner that the attaching transducer can receive an electrical signal. The thin layer of composite nanoparticles interacts with phthalimide, catalyze a reaction selectively, or take part in a chemical equilibrium along with the analyte. In the developed phthalimide sensor, the interaction process is adsorption which mainly performs at the boundary connecting analyte and receptor surface. The interaction between receptor and anlyte produce electrical signal by change in physical properties which later processed by tranducer (electrical instrumentation). This tranducer performs transducing function i.e, it transforms a non-electric quantity (the actual concentration value) into an electric quantity (current) [20], [21].

**Figure 5:**
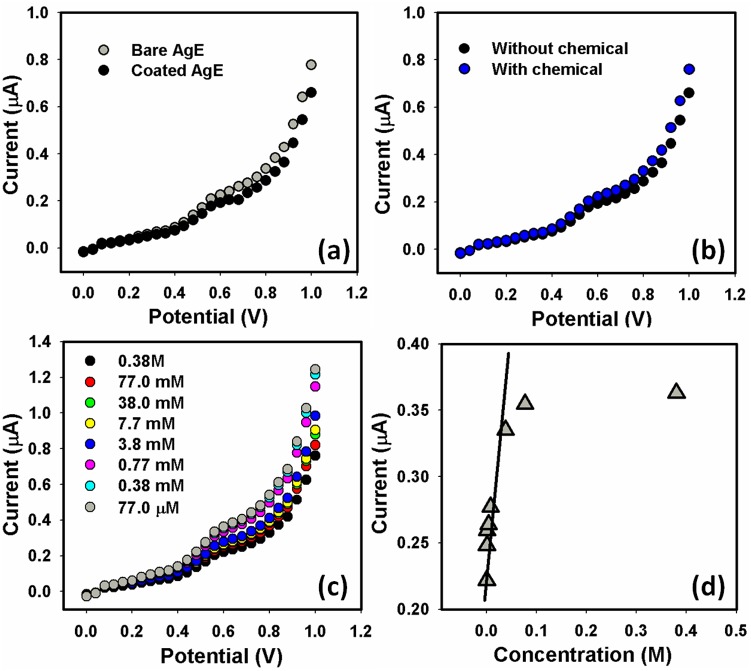
I–V characterization of composite nanoparticles (a) Comparison of with and without composite nanoparticles coating on AgE, (b) Comparison of with and without phthalimide, (c) effect of phthalimide concentration, and (d) calibration plot.

The electrical current of the developed sensor was studied and the electrical current of composite nanoparticles modified silver electrode (working electrode) were assessed in the presence of target chemical and shown in Fig. 5(a). Fig. 5(a) shows the electronic signal, being a current change is due to adsorption of phthalimide onto the surface of the sensor. Fig. 5(b) displays current of working electrode lacking phthalimide and with 100.0 µL phthalimide. Addition of phthalimide increases electrical current showing that composite nanoparticles are sensitive to phthalimide. Fig. 5 indicates that the developed chemical sensors transformed chemical information of phthalimide into an analytically valuable signal. The chemical information which causes signal originat either as a result of phthalimide reaction (chemical reaction) or from a physical property of the system investigated. Thus by insertion of phthalimide, augment in current imply that composite nanoparticles has quick response to the phthalimide. The developed chemical sensor send out energy signal in the form of electrical signal and interpret the change in property originated by the chemical [22]. The quick electron exchange and better electro-catalytic redox properties are also responsible for the quick electrical response of composite nanoparticles to phthalimide [23]. The change in resistance (electric current) of the composite nanoparticles takes place on exposing to phthalimide due to possible reaction between phthalimide and composite nanoparticles [22], [23].

#### 2.2. Influence of phthalimide concentration on sensor

The concentration effect of phthalimide on the electrical current of sensor was examined in the concentration of 1.0 µM to 0.05 M and the results are revealed in Fig. 5(c). The upshots display the dependence of electrical current on concentration of phthalimide. The current signals of the composite sensor are associated to the quantity of phthalimide by Faraday’s law and the laws of mass transport. It is working in an area where mass transport was restraining and as a result it has a linear response with concentration of phthalimide. The gradual increase in current also suggests that amount of ions rises with upturn in phthalimide concentration by providing additional electron to the conduction band of composite nanoparticles [18]–[23].

Calibration arc was designed from the current discrepancy and is illustrated in Fig. 5(d) The calibration arc indicate that in the beginning current increases with rise in phthalimide concentration but behind certain concentration, the current turn into constant which reflect permeation at this particular concentration. The lower portion of the calibration curve is linear having correlation coefficient (R) of 0.9673 whereas the slope represents sensitivity which is 1.7361 µA.cm^−2^.µM^−1^. Composite nanoparticles exhibited linear dynamic range from 77.0 µM to 0.38 M and detection limit of 8.0 µM. The linearity of composite nanoparticles represents the receptive region for phthalimide which specifies its sensitivity and detects phthalimide at micro level. The fabricated sensors can be useful at lower phthalimide concentration [22]. Fig. 5(d) indicates that exposing the sensor to the phthalimide, initially chemisorptions of phthalimide takes place on the surface and then further increase in phthalimide concentration cause physisorption. At lower phthalimide concentration, the sensor shows much increase in current which may be due to the donation of electron to composite nanoparticles by chemisorption and ionic conduction because of little absorption of phthalimide. At higher concentration, the electrical current becomes almost constant because as phthalimide concentration increases, the physiosorption start which might be due to condensation of phthalimide molecules on the surface of composite nanoparticles.

#### 2.3. Resistance behavior of phathalimide sensor

**Figure 6:**
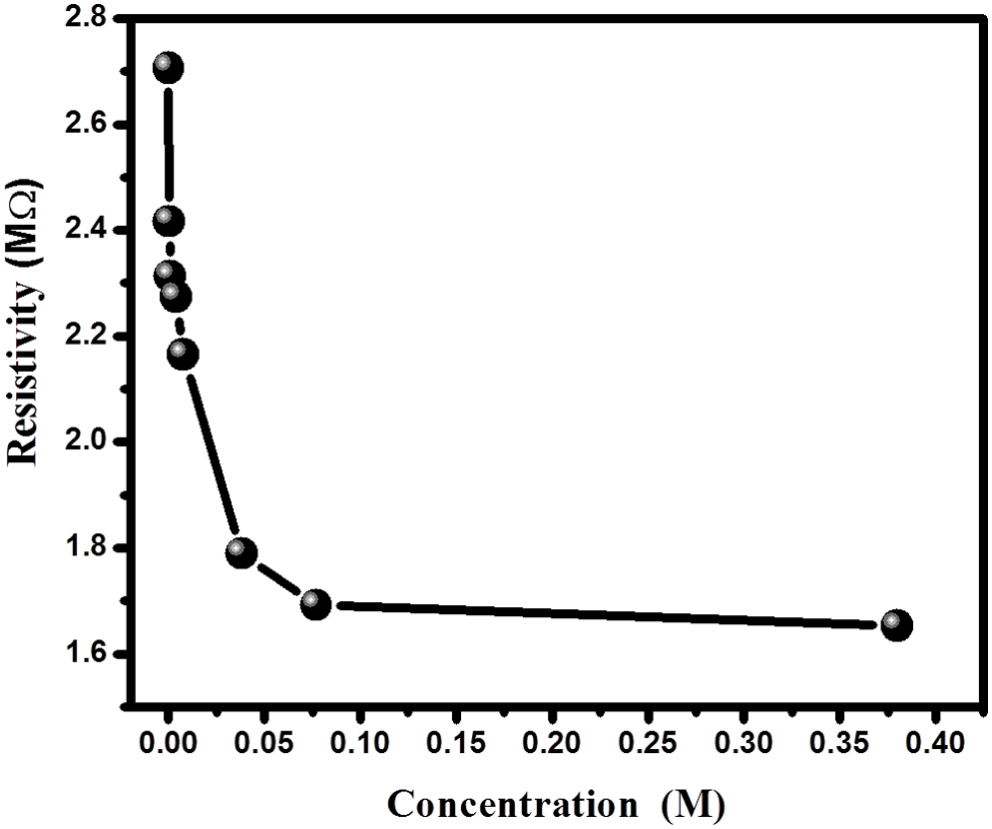
Resistivity of sensor toward phthalimide concentration.

The phthalimide resistance behavior of composite nanoparticles based sensor is shown in Fig. 6. It can be seen that the initial resistances of composite nanoparticles is 2.7 MΩ. The change in phthalimide concentration from 77.0 µM to 0.38 M causes change in resistance from 2.7 to 1.7 and become stable there after without further decrease. On exposing the sensor to the environment where phthalimide concentration increases gradually, in the start at lower phthalimide concentration, the chemisorptions of phthalimide takes place on the surface, while on further increase in phthalimide concentration, the physisorption takes place [10]. In the lower range of phthalimide, the sensors show large change in resistance. The reasons for large change in resistance may be the donation of electron to composite nanoparticles by ionic conduction and chemisorption of phthalimide. As phthalimide concentration increases, the physiosorption starts and phthalimide molecules start to condense on the surface of composite nanoparticles. During this stage initially chemisorbed and first physisorbed layers contribute in conduction by tunneling between donor phthalimide sites and electron hopping along the surface of composite nanoparticles, while the further condensation results in ionic conduction and the rate of change of resistance becomes small. There may be many other reasons for the increase in electrical current and decrease in resistance with increase in phthalimide concentration; which include absorption of phthalimide molecule in the composite nanoparticles (that owing to displacement current reduces the resistance and augments the current) [24]–[27].

#### 2.4. Advantages of phathalimide sensor

A huge number of sensors have now been developed for different analytes such as glucose, ethanol, ammonia, acetone, and many other chemical [1]. But the composite nanoparticles based chemical sensor is more beneficial as compared to several other types of sensors since it has trivial size, little power, extraordinary sensitivity, along with comparatively low price, assembling it perfect for handy toxic and volatile chemical equipment. It has been realized that decrease in size commence novel properties (electrical, mechanical, chemical, catalytic and optical properties) [1]. Composite nanoparticles can be used to fabricate sensors that identify extremely tiny quantity of phthalimide. Various types of nanomaterials have been previously utilized as a detecting element in nanotechnology-based sensors. Composite nanoparticles (detecting element) alter its electrical properties (resistance or capacitance) while they absorb phthalimide. Owing to the small size of composite nanoparticles, a little phthalimide is enough to alter electrical properties of composite nanoparticles (sensing element) [10]. This tolerates recognition of extremely tiny amount of target chemicals and this is the goal and requirement to have small and low cost sensors that can detect phathalimide at ultra trace level. Therefore, the composite nanoparticles are outstanding aspirant for the progress of competent and utmost sensitive phthalimide sensor and the consequent sensitive sensor for phthalimide will be helpful in medicinal and environmental applications [1], [10].

#### 2.5. Detection mechanism of phathalimide sensor

The detection mechanism and change in the electrical conductance can be explained on the bases of Henry and Peter model (microscopic model) [1], [24], [27]. This model explains the reactions occurring on the surface of composite nanoparticles in the existence of hazardous chemical.

**Figure 7:**
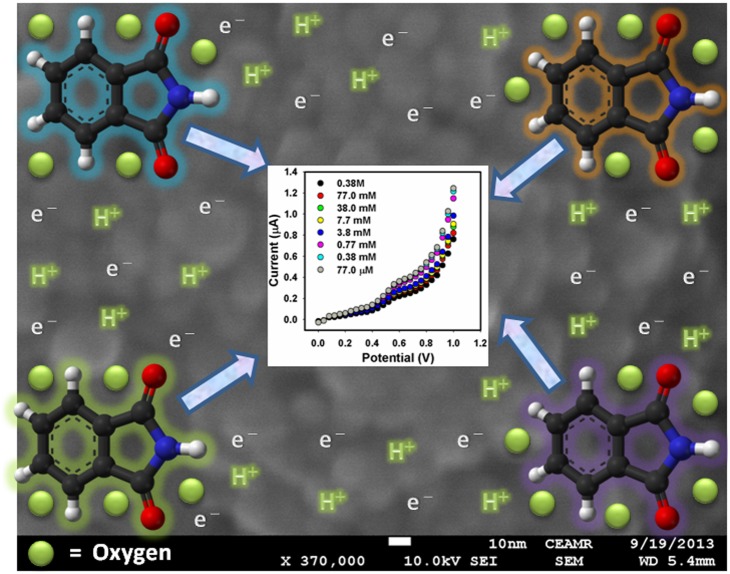
Schematic views of the reactions occurred at fabricated electrode for the recognition of phthalimide.

The relationship among the variation in resistance to different quantity of phthalimide possibly be explained by power-law equation. Composite nanoparticles based sensor reply to the variations in the concentration of phthalimide which alter the resistance of composite nanoparticles [27]. Initially, oxygen is physisorbed on top of the nanoparticles. The adsorbed oxygen gets ionized as O_ads_^−^ (O^−^ or O_2_^−^) by transferring the donor electrons to it and leaves positive charge in the composite layer [24], [27]. This phenomenon brings a reduction in the conductance of the transducer and an upsurge in potential barrier at the grain limits. O_ads_^−^ release the confined electron to the conduction band of composite nanoparticles and cause the fall of potential barrier at grain boundary. Further, when phthalimide react with already adsorbed negatively charged oxygen adsorbates, the electrons are returned to conduction band of the material (Fig. 7). The decay of adsorbed molecules releases some energy which would be ample for the electrons leaping up into the conduction band, consequently enhancing the conductivity of sensor [27]. In sensor, electrical current moves through the grain periphery of composite nanoparticles. Electrical current is opposed by negatively charged oxygen present at grain boundaries. A surface catalyzed reaction takes place in existence of a reducing chemical, decreasing the surface density of negatively charged oxygen, thus reducing the resistance of the sensor [24], [27].

## Conclusion

In the current study, composite nanoparticals were produced by using low temperature hydrothermal method. The comprehensive characterizations of composite nanoparticals were carried out by investigating its XRD, FESEM, FTIR, EDS, and UV-Vis spectra. All the analytical tools reveal that the composite nanoparticals are well-crystalline and optically active. The composite nanoparticals were utilized for the recognition and monitoring of phthalimide in aqueous media. The developed phthalimide sensor is based on change in resistance as well as electrical current. Investigations of the electric properties of sensor reveal that increase in concentration of phthalimide decreases the resistance and increases current which are attributed to the generation of charge carriers. The developed phthalimide sensor has high sensitivity, lower limit of detection and wide range of detection. The fabricated sensor would have great potential for the monitoring of toxic phthalimide chemical.
